# Transcranial Direct Current Stimulation Does Not Counteract Cognitive Fatigue, but Induces Sleepiness and an Inter-Hemispheric Shift in Brain Oxygenation

**DOI:** 10.3389/fpsyg.2018.02351

**Published:** 2018-11-30

**Authors:** Guillermo Borragán, Médhi Gilson, Carlos Guerrero-Mosquera, Eleonora Di Ricci, Hichem Slama, Philippe Peigneux

**Affiliations:** ^1^UR2NF, Neuropsychology and Functional Neuroimaging Research Unit, Centre de Recherches en Cognition et Neurosciences and UNI – ULB Neurosciences Institute, Université Libre de Bruxelles (ULB), Brussels, Belgium; ^2^Center for Brain and Cognition, Universitat Pompeu Fabra, Barcelona, Spain; ^3^UNESCOG – Cognitive Neurosciences Research Unit, Center for Research in Cognition and Neurosciences, Université Libre de Bruxelles (ULB), Brussels, Belgium; ^4^Department of Clinical and Cognitive Neuropsychology, Erasme Hospital, Brussels, Belgium

**Keywords:** cognitive fatigue, tDCS, fNIRS, inter-hemispheric balance, sustained attention, sleepiness

## Abstract

Sustained cognitive demands may result in cognitive fatigue (CF), eventually leading to decreased behavioral performance and compromised brain resources. In the present study, we tested the hypothesis that transcranial direct current stimulation (tDCS) would counteract the behavioral and neurophysiological effects of CF. Twenty young healthy participants were tested in a within-subject counterbalanced order across two different days. Anodal tDCS (real vs. sham) was applied over the left prefrontal cortex. In the real tDCS condition, a current of 1.5 mA was delivered for 25 min. Cortical oxygenation changes were measured using functional Near Infrared Spectroscopy (fNIRS) on the frontal cortices. CF was triggered using the TloadDback task, a sustained working memory paradigm that allows tailoring task demands according to each individual’s maximal cognitive capacity. Sustained cognitive load-related effects were assessed using pre- versus post-task subjective fatigue and sleepiness scales, evolution of performance accuracy within the task, indirect markers of dopaminergic activity (eye blinks), and cortical oxygenation changes (fNIRS) both during the task and pre- and post-task resting state periods. Results consistently disclosed significant CF-related effects on performance. Transcranial DCS was not effective to counteract the behavioral effects of CF. In the control (sham tDCS) condition, cerebral oxygen exchange (COE) levels significantly increased in the right hemisphere during the resting state immediately after the induction of CF, suggesting a depletion of brain resources. In contrast, tDCS combined with CF induction significantly shifted interhemispheric oxygenation balance during the post-training resting state. Additionally, increased self-reported sleepiness was associated with brain activity in the stimulated hemisphere after recovery from CF during the tDCS condition only, which might reflect a negative middle-term effect of tDCS application.

## Introduction

Cognitive fatigue (CF) can be defined as temporary compromised mental resources developing over time on sustained cognitive demands/effort. The onset of CF is gradual and depends on each individual’s capacity. Its presence often comes with an increased subjective feeling of mental exhaustion and a usual failure to maintain optimized behavioral performance (Borragán et al., 2017b). CF is responsible for decreased behavioral performance and increased propensity to errors (Boksem et al., 2005; Lorist et al., 2005) following sustained cognitive demands. This makes it a topic of interest both in experimental and ecological settings. Indeed, CF represents an important confound in experimental contexts (Ackerman, 2011) and can be a life-threatening factor in working populations (Dawson and Fletcher, 2001) as well as a contributing factor in major accidents (e.g., Chernobyl, Three Mile Island or Bhopal disasters; Turner, 2012). Consequently, various countermeasures have been investigated to prevent or limit the negative effects of CF. For instance, optimizing work schedules was shown to reduce CF (Rose and Curry, 2009), whereas amphetamines (Ilieva et al., 2015; Spencer et al., 2015) and caffeine (Lorist and Tops, 2003; Van Duinen et al., 2005; McIntire et al., 2014; Urry and Landolt, 2015) stimulants can improve cognitive functioning on the short term and delay the development of CF. Transcranial DCS (tDCS) is a non-invasive electrical brain stimulation technique that increases or decreases neuronal excitability in superficial cortical areas. As an example, 5 min of anodal tDCS can induce substantial cortical excitability changes, that may last for several hours after the actual stimulation period (Bindman et al., 1962; Merzagora et al., 2010). Literature describes anodal tDCS as a potential performance enhancer, with the potential to enhance attention, learning, and memory (Coffman et al., 2014). For instance, anodal tDCS over the left dorsolateral prefrontal cortex significantly improved target detection, and delayed the natural decrease of blood flow velocity within time-on-task (Nelson et al., 2014). Likewise, anodal tDCS was shown to prevent vigilance decrement and increased fatigue, drowsiness and lack of energy during sleep deprivation (McIntire et al., 2014). Consequently, tDCS was seen as a potential instrument to improve cognitive functioning both in healthy (Kuo and Nitsche, 2012, for a review) and neurological populations (Flöel, 2014, for a review). Yet, variability is high among studies and participants (Kim et al., 2014), and positive effects have been questioned (Horvath et al., 2015b) or reported to be beneficial in specific conditions, e.g., for low performers only (Tseng et al., 2012).

In the present study, we tested whether tDCS can counteract the development of CF during and after a cognitively demanding dual working memory paradigm, the TloadDback task (Borragán et al., 2017b). Working memory paradigms such as the N-Back or the TloadDback tasks have been traditionally used to induce high levels of sustained cognitive demands mostly relying on fronto-parietal activity (Owen et al., 2005; Herff et al., 2014; León-Domínguez et al., 2015). The TloadDback presents the particularity to adapt the level of cognitive demands to each participant’s maximal processing capacity, therefore limiting the impact of inter-individual differences. TloadDback was shown robust to induce subjective fatigue and a decrease in performance over time during task practice (Borragán et al., 2016, 2017a,b). To track the evolution of CF in relation with tDCS vs. Sham [control] conditions, we used a multidimensional approach assessing behavioral, subjective and physiological markers of CF. At the behavioral level, the development of CF and vigilance was estimated using pre vs. post task CF questionnaires and performance on the canonical psychomotor vigilance task (PVT; Dinges and Powell, 1985; Basner and Dinges, 2011), and by measuring the evolution of performance during TloadDback practice. At the neurophysiological level, tDCS- and CF-related changes in cortical oxygenation during the TloadDback task and in the subsequent resting state were estimated using functional Near Infrared Spectroscopy (fNIRS). Functional NIRS is a non-invasive technique that takes advantage of light diffusion properties in nearby tissues to track hemoglobin oxygenation in cortical brain regions (Ferrari and Quaresima, 2012). In line with prior reports (e.g., McIntire et al., 2014; Nelson et al., 2014), we hypothesized that tDCS application over the left dorsolateral prefrontal cortex (DLPFC) during task practice would reduce the feeling of CF eventually delaying the time-related decrease in task performance observed in studies using a comparable paradigm (Shaw et al., 2009; Borragán et al., 2016, 2017b). Furthermore, we hypothesized that tDCS stimulation would counteract the known decrease in frontal oxygenation during a resting state period following the induction of CF (Lim et al., 2010). Additionally, spontaneous eye blinks and yawning, taken as indirect markers of dopaminergic activity associated with the triggering of CF (Karson, 1983; Stern et al., 1994; Walusinski and Deputte, 2004), were recorded during the immediate pre- and post-task resting state periods. Finally, we tentatively probed potentially delayed effects of tDCS on the recovery of CF. Indeed, if an immediate impact of tDCS is well documented (see e.g., for review Matsumoto and Ugawa, 2017), less is known about potential medium terms effects.

## Materials and Methods

### Subjects

The optimal sample size to test the desired fatigue effect was computed using a statistical power analysis (G^∗^Power 3.1.7; Faul et al., 2007). *Partial*-η^2^ values disclosed in the experiments presented in Borragán et al., 2017b (η^2^ = 0.3 and 0.4) for the main effect of Time on Task indicated a required sample (*N*) of at least 16 subjects [effect size = 0.73, power (1−β) = 0.95)]. Twenty-two right-handed healthy young adult participants (mean age ±*SD* = 23 ± 2.28 years; 8 men) gave their informed consent to participate in the present study conducted in agreement with the Declaration of Helsinki and approved by the Faculty Ethics Committee of the Université Libre de Bruxelles and Erasme hospital (*N*° = 021/406). Participants were naïve about the purpose of the experiment, and received a monetary compensation of 40€ for their participation. Exclusion criteria were bad sleep quality [global score > 7 at the Pittsburgh Sleep Quality Index (Buysse et al., 1989)], moderate to severe levels of usual CF [cognitive score > 28 at the Fatigue Scale for Motor and Cognitive Functions (Penner et al., 2009)], excessive sleepiness [Epworth Sleepiness Scale score > 10 at the (Johns, 1991)] over the last month, and excessive levels of anxiety and depression [Hospital Anxiety and Depression Scale score > 10 (Zigmond and Snaith, 1983)]. Two subjects did not complete the last session on day 3; their data were excluded from the analyses. Supplementary Table S1 reports mean (± standard deviations) scores on these scales. To control for the regularity of sleep-wake activity during the experiment, participants wore an actigraphy recording device (ActiGraph, wGT3X-BT Monitor, United States) at the non-dominant wrist during the 3 days of the experiment (movement values summarized over 10-s periods), and completed at the beginning of each session a questionnaire (QSN; St-Mary Hospital Questionnaire, Ellis et al., 1981) about their past night of sleep (duration, quality, awakenings…).

### Measures: Experimental Tasks

#### Cognitive Fatigue Induction: TloadDback

Cognitive fatigue was triggered using the TloadDback task (Borragán et al., 2016, 2017b) that combines in a dual setting an updating working memory task (N-back; Kirchner, 1958) and a parity number decision task. More precisely, 30 digits and 30 letters per block are displayed on screen in alternation, and blocks are repeated during the 16-min duration of the task. Participants are instructed to press the space bar with their left hand every time the displayed letter is the same than the penultimate letter, or to indicate whether the displayed digit is odd or even by pressing “1” or “2” on the numeric keypad. Combining two tasks featuring different processing requirements is aimed at ensuring a large recruitment of working memory resources, which was shown to lead to decreased performance and increased feelings of mental exhaustion (Borragán et al., 2016, 2017b). In the pre-test session on Day1, the maximal load level [i.e., the fastest stimulus time duration (STD) allowing accuracy performance > 85%] was determined separately for each participant in a horse race procedure (i.e., STD was made faster by 100 millisecond-steps until performance accuracy within a 60-trials block dropped below 85%); the fastest STD with >85% accuracy performance was then used for the subsequent 16-min administrations (held on the next days) of the TloadDback task to this same individual. Task-related changes in CF were assessed (a) at the subjective level using the Visual Analog Scale for fatigue (VASf; Lee et al., 1991; see below) before and after the TloadDback task, and (b) objectively by computing the evolution of performance accuracy within the TloadDback task over four successive 4-min duration time periods (t1, t2, t3, and t4).

#### Vigilance, Eyeblink and Yawning Rates

During the three resting-state fNIRS recording (see below) sessions, participants were asked to keep their eyes open and to fixate a cross on the center of the computer’s screen, for a total duration of 4 min. During this period, the participant’s face was video-recorded to allow ulterior quantification of the number of eye blinks (eyeblink rate) and yawns (yawning rate). Immediately after each resting state session, participants were administered the 5-min version of the Psychomotor Vigilance Task (PVT-5; Dinges and Powell, 1985; Basner and Dinges, 2011). In this task, a visual timer starts at random intervals (ranging 2–10 s); participants are instructed to stop the timer as fast as possible by pressing the space key. A feedback reaction time is provided at each trial. Reciprocal reaction times (1/RT) were computed as the most sensitive measure of arousal levels in the PVT (Basner and Dinges, 2011).

#### Subjective Assessment of Cognitive Fatigue (CF) and Sleepiness

Subjective CF was evaluated using the Visual Analogic Scale of Fatigue (VASf; Lee et al., 1991). During this assessment, participants were given a paper with a 12 cm horizontal line which represented potential variations in their level of subjective CF, from none at all (left) to extreme (right). They were asked to cross the horizontal line to indicate the level of fatigue that they felt at that specific time. Importantly, answers made at previous evaluations were not visible to the participant to avoid visual comparison biases. Subjective sleepiness, which is a distinct concept of CF (Neu et al., 2010), was assessed using a similar Visual Analogic Scale of Sleepiness (VASs) adapted from the Epworth Sleepiness Scale (Johns, 1991).

### Brain Stimulation (tDCS) and fNIRS Recordings

#### Transcranial Direct Current Stimulation (tDCS)

**FIGURE 1:**
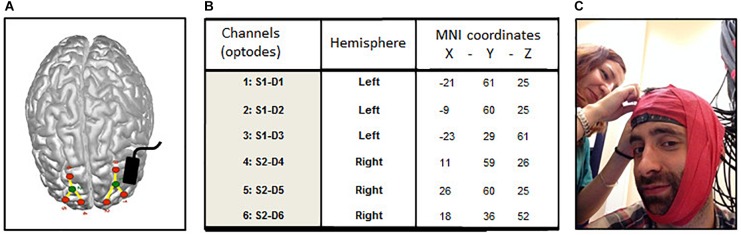
Localization of optodes (fNIRS) and anode (tDCS). **(A)** Averaged emplacement of the NIRS optodes (green: source, red: detectors) and tDCS anode (black rectangle) projected on the MNI brain template. **(B)** Averaged MNI coordinates of the 6 NIRS channels (detectors). **(C)** Elastic red tape is used to prevent shifts in channel location during the experiment.

Transcranial direct current stimulation (tDCS) was provided using a NeuroConn DC stimulator (DC-Stimulator Plus, NeuroConn, Ilmenau, Germany). Following the setup of prior studies (McIntire et al., 2014; Nelson et al., 2014), a current of 1.5 mA was delivered for 25 min between two 5 cm × 5 cm conductive rubber electrodes in the real tDCS condition (Figure 1). For safety reasons and to control for the correct functioning of the device, continuous monitoring of the output current was performed. The anode was positioned over the left DLPFC (F3 location according to the 10–20 EEG electrode coordinates system using the tDCS GI Placement System) and the cathode was positioned over the right forearm in line with prior studies (McIntire et al., 2014). Electrodes were fixed using elastic bandages and connectivity was improved using a saline solution (Ten20; MedCat B.V). In the sham tDCS condition, stimulation ascended during a 10 s ramp up to 1.5 mA, then stabilized for 30 s and then gradually faded out to 0 mA throughout the next 10 s at the beginning of the task. Total stimulation time during this Sham condition was thus 50 s. Participants were blind with respect to the condition of stimulation (real versus sham tDCS).

#### fNIRS Recording

The effect of tDCS upon brain cortical activity was assessed using a multichannel fNIRS system (BrainSight V2.3b16, Rogue Research Inc., Canada) with two continuous wavelengths of 685 and 830 nm. The set-up of the optodes included 2 sources and 6 detectors per hemisphere, for 3 channels located over superior frontal cortices across cortical locations (see Figure 1A) previously shown to be involved in working memory (Barbey et al., 2013) and sustained attention (McIntire et al., 2014; Nelson et al., 2014) and to be active in the resting state (Raichle, 2013). Detector optodes were positioned at a distance of 3 cm from the source optodes using a 3-D coordinates system combined with a Polaris localization device (averaged MNI coordinates Figure 1B). Optodes’ shift during the experiment was prevented using elastic red tape (Figure 1C). NIRS raw signals were digitized at a sample rate of 20 Hz. For the analyses, the signals of the three channels within each hemisphere were averaged to provide a general measure of brain activity. For each participant, raw recorded absorption units were normalized then low-pass filtered (0.009–0.08 Hz) to attenuate high-frequency noises arising from respiration, cardiac pulsations and optodes’ movements. Homer toolbox functions were employed for filtering and optical density computations (Huppert et al., 2009). The resulting signals were then converted into their hemoglobin oxygenated (HbO) and de-oxygenated (HbR) components using the modified Beer-Lambert law (Delpy et al., 1988). Event’s onset and offset (in the TloadDback task) were individually triggered to obtain accurate times duration. Grand average of brain activity changes per time period during the TloadDback task (by 4-min blocks during the 16 min; t1–t4) and during the 4 min Rst sessions were calculated using cerebral oxygen exchange (COE) measures. COE provides an indirect measure of brain metabolism and it is computed as the difference between deoxygenated (HbR) and oxygenated (HbO) hemoglobin in the tissue at a specific time point (COE = HbR – HbO; Yoshino et al., 2013). Negative COE values indicate increased cortical oxygenation whereas positive values represent hypoxic changes.

### Experimental Design

**FIGURE 2:**
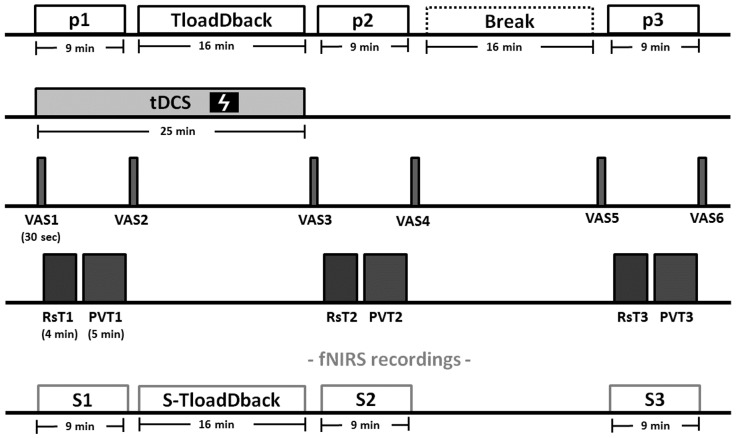
Experimental procedure (Days 2 and 3). Each evaluation period (p1, p2, and p3) comprises (1) visual analog scales for fatigue (VASf) and sleepiness (VASs), (2) a 4-min resting state (Rst) period, (3) a 5-min version of the psychomotor vigilance task (PVT) and (4) again the VASf and VASs. After the evaluation period p1, the CF-inducing TloadDback was administered for 16 min, immediately followed by the second evaluation period (p2). The third evaluation period (p3) was then administered after a 16-min break (recovery time). Brain activity was recorded using fNIRS during the 3 evaluation periods and the TloadDback task. Additionally, eyeblink and yawns were video recorded during the resting state sessions. tDCS (sham or real, in counterbalanced order) was applied for 25 min starting from the beginning of the experiment.

The experiment was conducted over three consecutive days in a within-subject counterbalanced design (see Figure 2 for an overview of the experimental procedure). On Day 1, cognitive load levels were individually adjusted during a pre-test practice session on the TloadDback task, i.e., we determined for each participant in a horse race procedure the fastest presentation time (thus maximal cognitive load) allowing to keep performance accuracy above 85% (Borragán et al., 2017b). The pre-test procedure allows tailoring task demands according to each individual’s cognitive capacity, eventually inducing comparable levels of CF between participants. On Days 2 and 3, the TloadDback task was administered at the individual’s maximal cognitive load level (i.e., fastest STD determined in the calibration session at Day 1), either under a real or a sham tDCS (see below) stimulation condition, counterbalanced. Each session on Days 2 and 3 started with setting up the tDCS device and the fNIRS equipment (see below). Real or Sham tDCS was then applied for 25 min. During the actual stimulation period, participants completed the first evaluation period (p1) and the TloadDback task. For the first evaluation period, participants filled in the subjective visual analog scales (VAS1) for CF (VASf; Lee et al., 1991) and sleepiness (VASs; Johns, 1991) followed by a 4-min fNIRS acquisition in the resting state, eyes open, (Rst1) and then a 5-min version of the Psychomotor Vigilance Task (PVT1; Dinges and Powell, 1985; Basner and Dinges, 2011), then completed again the VASf and VASs (VAS2). The TloadDback task was then performed for 16 min with cortical activity recorded using fNIRS, after what tDCS was switched off. A second testing period (p2), identical to p1, was then administered (VAS3, fNIRS Rst2, PVT2, and VAS4), followed by a recovery time (Break) during which participants were allowed a light reading activity (magazines and comics) for the same duration (16 min) than the TloadDback task. A third testing period (p3; identical to p1 and p2) was then administered (VAS5, fNIRS Rst3, PVT3, and VAS6). Additionally, yawning and eye blinking rates were video-recorded during the three 4-min resting state (Rst) sessions.

#### Statistics

Statistical analyses were computed following Fritz et al. (2012) recommendations. Mean (*m*) ± Standard Deviation (*std*) are reported as measures of central tendency, and size effects are reported as partial eta squares (η^2^). Mean squared errors (*MSE*) are included in the ANOVAs. Significance level was set at *p* < 0.05 (two-tailed) and Tukey HSD test were employed for *post hoc* corrections. Bayes factors (BF) were additionally computed where needed (JASP-software; Love et al., 2015), considering that BF values > 3 are considered as substantial evidence for the alternative hypothesis (H1), BF values < 0.333 indicate substantial evidence for the null hypothesis (H0), and values between 0.333 and 3 are deemed inconclusive and indicate a lack of sensitivity (Dienes, 2011).

## Results

### Sleep-Wake Cycle Regularity and Sleep Quality

Separate repeated-measure ANOVA were computed on subjective sleep quality scores (from 1 [very bad] to 6 [very good]) and sleep duration (hours) as derived from the QSN (Ellis et al., 1981) for the three nights preceding the experimental days, with Night as within-subject factor. The effects were non-significant in both ANOVAs (*p*s > 0.17), showing that Sleep quality (Night1 = 4.7 ± 0.098, Night2 = 5.1 ± 0.97, and Night3 = 4.9 ± 0.83) and sleep duration (Night1 = 7.53 ± 1.3, Night2 = 7.2 ± 1.16, and Night3 = 7.3 ± 1.6 h) were similar for the three experimental nights covering the TloadDback pretest and the counterbalanced Sham and Real tDCS sessions.

Inspection of individual actigraphic recordings confirmed self-reported sleeping and waking up hours and the regularity of the sleep-wake cycle. Additionally, a repeated-measure ANOVA computed on hourly averaged actimetric activity values over the day (16 h) and night (8 h) periods, with Cycle (3 consecutive days) and Moment (Night vs. Day) as within-subject factors, disclosed a main effect of Moment [*F*_(1,19)_ = 59; *p* < 0.001; *MSE* = 1.7e+09; *partial*-η^2^ = 0.84] with higher motor activity within the day than the night, as expected in a day wake – night sleep activity pattern. No other main effect or interaction reached significance (all *p*s > 0.1). Altogether, these results suggest that participants got sleep at night and respected a regular sleep-wake schedule during the experiment.

### Behavioral and Cognitive Performance

#### Subjective Ratings of CF and Sleepiness

**FIGURE 3:**
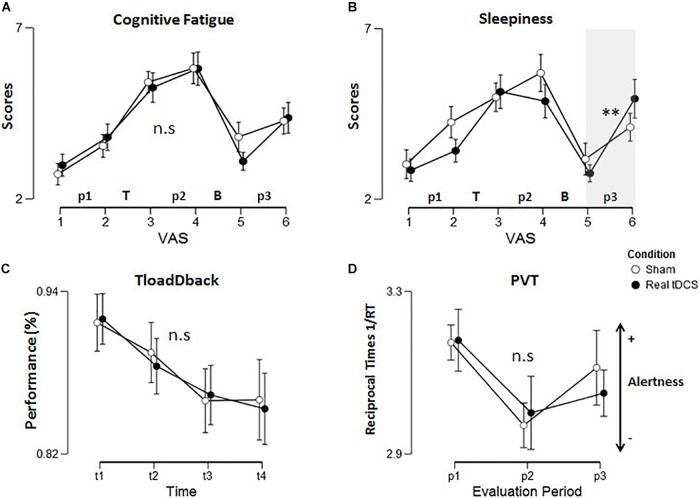
Behavioral data. Self-reported **(A)** cognitive fatigue and **(B)** sleepiness during the six VAS assessment points in the experiment. “T” refers the TloadDback task and “B” the recovery break. **(C)** Performance (accuracy) changes during practice of the TloadDback task (across 4-min quartiles). Performance significantly decreased with time on task from t1 to t3 then stabilized (t1 > t2 > t3 = t4). **(D)** Evolution of vigilance (reciprocal 1/RT) across the three evaluation periods. Lower 1/RT values indicate lower vigilance levels. Error bars are standard errors. Asterisks reflect *p*-values after Tukey *post hoc* correction: ^∗^*p* < 0.05, ^∗∗^*p* < 0.01, ^∗∗∗^*p* < 0.001. n.s: non-significant.

First, we tested whether CF and sleepiness measurements remained stable across the two repeated measurements at each of the three evaluation periods (p1, p2, and p3). To do so, differences in VASf or VASs (before vs. after the Rst/PVT; i.e., VAS2 minus VAS1 for p1, VAS4 minus VAS3 for p2, and VAS6 minus VAS5 for p3; see Figure 1) were computed for each period, then introduced in a repeated-measure ANOVA with Evaluation Period (p1 vs. p2 vs. p3) and Condition (Sham vs. Real tDCS) as within-subject factors. Results disclosed a similar evolution of CF in Sham and Real tDCS conditions during the three evaluation periods (all *ps* > 0.3; BFs < 0.33; Figure 3A). The same ANOVA conducted on sleepiness scores (VASs) disclosed a main effect of the Evaluation Period [*F*_(2,38)_ = 3.88; *p* < 0.05 *MSE* = 4.64; *partial*-η^2^ = 0.17] with a steeper increase in sleepiness during the p3 period (1.56 ± 1.29) than p1 (.22 ± 1.80; *p* < 0.05). Results also disclosed an Evaluation Period × Condition interaction effect [*F*_(2,38)_ = 3.23; *p* = 0.050 *MSE* = 4.55; *partial*-η^2^ = 0.14]. *Post hoc* tests showed that sleepiness scores increased significantly after the Break during the p3 evaluation period in the Real tDCS (*p* < 0.01) but not in the Sham condition (*p* > 0.99; Figure 3B).

Second, we looked at the effect of TloadDback practice and of the subsequent recovery period on the evolution of CF and sleepiness. Pre- vs. post-task practice (VAS3 minus VAS2) and recovery period (VA5 minus VAS4; see Figure 1B) differential scores were entered in a repeated measure ANOVA with Intervention Period (TloadDback vs. Break) and Condition (Sham vs. Real tDCS) as within-subject factors. The ANOVA conducted on CF (VASf) scores disclosed a main Intervention Period effect [*F*_(1,19)_ = 22; *p* < 0.001; *MSE* = 14.29; *partial*-η^2^ = 0.54]. CF increased after the TloadDback task (1.65 ± 1.86) then decreased again after the Break (−2.35 ± 2.14). No other effects were significant (*ps* > 0.14; BFs for Condition and Intervention Period < 0.33, BF for interaction = 0.37; Figure 3A). The ANOVA conducted on sleepiness scores (VASs) disclosed a main effect of the Intervention Period [*F*_(1,19)_ = 24; *p* < 0.001; *MSE* = 10.52; *partial*-η^2^ = 0.56]. Likewise, sleepiness increased after the TloadDback task (1.23 ± 1.4) then decreased after the Break (−2.32 ± 2.15; Figure 3B). Other effects were no significant (*p*s > 0.25; BF < 0.51).

#### Vigilance Levels (PVT)

A repeated measures ANOVA computed on reciprocal reaction times (1/RT; Basner and Dinges, 2011) with within-subject factors Evaluation Period (p1 vs. p2 vs. p3) and Condition (Sham vs. tDCS) disclosed a main effect of the Evaluation Period [*F*_(2,38)_ = 16; *p* < 0.001; *MSE* = 0.023; *partial*-η^2^ = 0.45]. Tukey *post hoc* tests disclosed decreased arousal (i.e., lower 1/RT) after the TloadDback task, from p1 to p2 (*p* < 0.001), then increased arousal after the recovery time, from p2 to p3 (*p* < 0.05). However, 1/RT at p3 was not back to baseline values (p1 > p3; *p* < 0.05; Figure 3D). Condition and interaction effects were non-significant (*p*s > 0.25|*BF*s < 0.33).

#### TloadDback Performance

A repeated-measure ANOVA conducted on accuracy scores (Figure 3C) in the TloadDback with Time on Task (first to fourth time quartile during practice; t1 vs. t2 vs. t3 vs. t4) and Condition (Sham vs. Real tDCS) as within-subject factors disclosed a main effect of Time on Task [*F*_(3,57)_ = 13; *p* < 0.001; *MSE* = 0.0025; *partial*-η^2^ = 0.4]. Tukey *post hoc* analyses evidenced decreasing accuracy during the first half of task practice (t1 > t2 > t3) then stabilization (t3 = t4). Condition and interaction effects were non-significant (Sham vs. Real tDCS; *p*s > 0.4). All Bayesian factors (BFs) were < 0.33, in favor of the null hypothesis (Love et al., 2015). Additional analyses computed separately for the digits and letters components of the TloadDback (Borragán et al., 2017b) gave similar non-significant condition and interaction effects (*all p*s > 0.9, *BF*s < 3).

#### Eyeblink and Yawning Rates

Spontaneous eye blinks and yaws during the 4-min resting-state periods were counted on the video recording as indirect physiological CF-related markers of dopamine level (Karson, 1983; Stern et al., 1994; Walusinski and Deputte, 2004). Separate repeated-measures ANOVAs were computed on eyeblink rate (EBR) and yawning rates with Condition (Sham vs. tDCS) and Evaluation Period (p1 vs. p2 vs. p3) as within-subject factors. Data are illustrated in Supplementary Figure S1. The results yielded a main effect of Evaluation Period for eyeblink [*F*_(3,57)_ = 6.3; *p* < 0.01; *MSE* = 364.2; *partial*-η^2^ = 0.25] and yawning [*F*_(3,57)_ = 6; *p* < 0.01; *MSE* = 0.49; *partial*-η^2^ = 0.25] rates. Tukey *post hoc* tests showed increased EBR after the TloadDback task [p1 > (p2 = p3); *p* < 0.05; p1 = 69 ± 45, p2 = 82 ± 53, and p3 = 83 ± 57]. As well, the yawning rate (p1 = 0.13 ± 0.48, p2 = 0.66 ± 0.86, and p3 = 0.24 ± 0.43) increased after the TloadDback task, from p1 to p2 (*p* < 0.01), but subsequently decreased over the recovery time from p2 to p3 (*p* < 0.05), to reach baseline levels (p1 = p3; *p* > 0.79). No other main effect or interaction was significant (eyeblink rate all *p*s > 0.17| all *BF*s < 0.33; yawning rate all *p*s > 0.12|*BF for Period* < 0.33, *BF for Period* × Condition = 0.49).

### Cortical Oxygenation (fNIRS)

NIRS recordings for one participant were very noisy and not usable. Thus, fNIRS analyses are conducted on 19 participants only.

#### Time on Task-Related Changes in Cortical Oxygenation

A repeated-measures ANOVA was computed on COE levels during the TloadDback task with ToT (first to fourth quartile during task practice; t1 vs. t2 vs. t3 vs. t4), Condition (Sham vs. Real tDCS) and brain Hemisphere (Left vs. Right) as within-subject factors. The analysis disclosed a main effect of ToT [*F*_(1,18)_ = 3.7; *p* < 0.05; *MSE* = 7.6e–11; *partial*-η^2^ = 0.17] with increased COE (i.e., decreased oxygenation) at the end of the task [t1 > t3; *p* < 0.02; t3 = t4; *p* > 0.59]. No other effects were significant (*p* > 0.16).

#### Hemispheric Cortical Oxygenation Changes Between Resting State Periods

Cerebral oxygen exchange values (see Table 1) were entered in a repeated-measure ANOVA with Evaluation Period (Rst1 vs. Rst2 vs. Rst3), Condition (Sham vs. Real tDCS) and Hemisphere (Left vs. Right) as within-subject factors. Results yielded a main effect of Condition [*F*_(1,18)_ = 18.52; *p* = 0.001; *MSE* = 8.4e–12; *partial*-η^2^ = 0.51] with globally higher oxygenation levels (i.e., lower COE) during the Sham than the Real tDCS condition and an Evaluation Period × Condition interaction effect [*F*_(2,36)_ = 6.64; *p* = 0.005; *MSE* = 1.02e–11; *partial*-η^2^ = 0.27]. *Post hoc* analyses disclosed significant differences between conditions at the third evaluation period (Rst3; *p* < 0.001) with lower oxygenation levels in the Real tDCS than in the Sham condition. Besides, oxygenation levels increased significantly from Rst1 to Rst3 in the Sham condition (*p* < 0.05).

**Table 1:** Cerebral Oxygen Exchange (COE) values between conditions within resting states.

Evaluation period	Condition	Mean	Standard error
Rst1	Sham	−3.75 × l0^14^	4.48 × l0^14^
Rst1	tDCS	−3.17 × l0^14^	5.52 × l0^14^
Rst2	Sham	−4.41 × l0^14^	3.39 × l0^14^
Rst2	tDCS	−4 × l0^14^	4.24 × l0^14^
Rst3	Sham	−6.17 × l0^14^	2.68 × l0^14^
Rst3	tDCS	−2.45 × l0^14^	3.95 × l0^14^

*Negative COE values indicate increased oxygenation whereas positive values represent hypoxic changes.*

**FIGURE 4:**
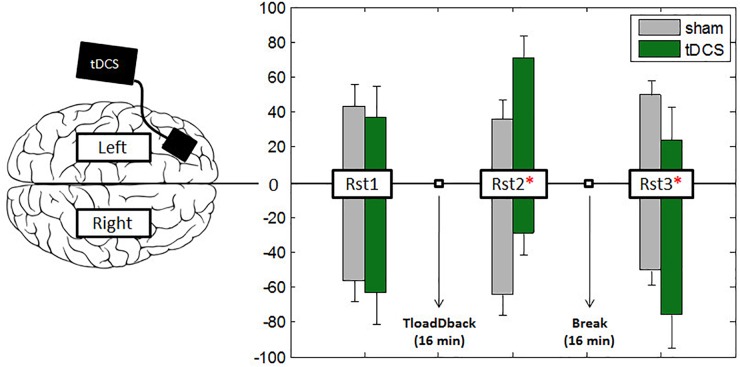
Asymmetric interhemispheric activation between conditions. Bars represent the percentage of total activity per hemisphere during the three resting states. Participants started the experiment with a 4-min resting state (Rst1) session followed by the 16-min performance with the TloadDback task. Right after they followed two more 4-min resting states (Rst2–Rst3) sessions. To test the middle-term effects of tDCS, a 16-min recovery period was introduced between resting states Rst2 and Rst3. A value of –100 in the *y*-ax indicates pure right-hemispheric dominance, and a value of +100 pure left-hemispheric dominance. Vertical bars represent standard errors and asterisks represent the existence of a significant asymmetry between hemispheres.

**FIGURE 5:**
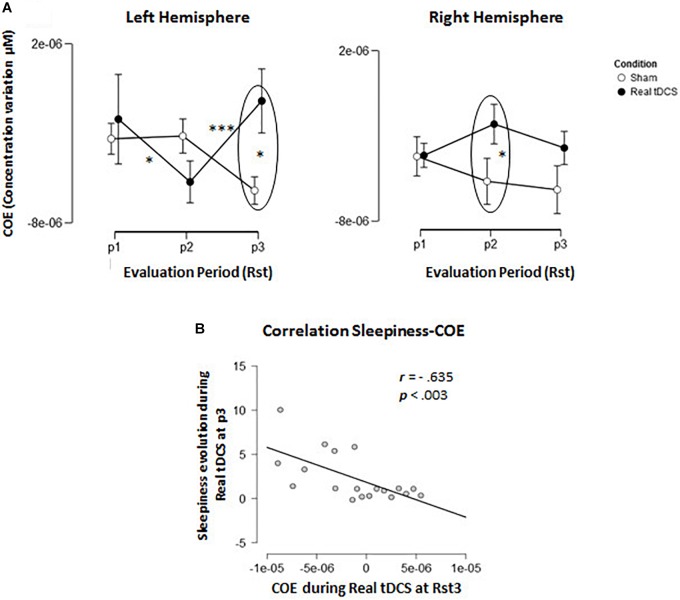
Cerebral oxygenation exchange (COE). **(A)** Evolution of delta COE during Conditions across the 3 resting state sessions in the left and right hemisphere. **(B)** Correlation-plot between COE levels at resting state 3 (RSt3) in the left hemisphere and evolution of sleepiness during the same period.

Furthermore, there was a triple Evaluation Period × Condition × Hemisphere interaction effect [*F*_(2,36)_ = 11.97; *p* = 0.001; *MSE* = 8.12e–12; *partial*-η^2^ = 0.4]. In the left hemisphere (where tDCS was positioned), Tukey *post hoc* tests showed that oxygenation levels increased significantly from Rst1 to Rst2 for the Real tDCS condition (*p* < 0.05) then decreased from Rst2 to Rst3 (*p* < 0.001). Oxygenation levels at Rst3 were also higher in the Sham than the Real tDCS condition (*p* < 0.001; see Figures 4, 5A). In the right hemisphere, Tukey *post hoc* tests disclosed a different evolution of brain activity between conditions. Oxygenation levels were significantly higher in the Sham than the Real tDCS condition at Rst2 (*p* < 0.05) but again similar following the recovery period at Rst 3 (see Figures 4, 5A).

#### Inter-Hemispheric Correlations and Relationships Between Sleepiness and COE

In light of these results, we investigated the relationships between increased self-reported sleepiness in the Real tDCS condition during Rst3 and the variations in oxygenation observed during the same period. Pearson correlation coefficients were computed between self-reported sleepiness and oxygenation changes in the right and left hemispheres separately. The results disclosed a negative relation (*r* = −0.635; *p* < 0.005/*p-value corrected for multiple comparisons; see* Figure 5B) between COE and sleepiness feelings only in the left hemisphere (*r* = −0.119; *p*-value > 0.62 in the right hemisphere).

## Discussion

The present study tested the hypothesis that tDCS over the left prefrontal cortex would counteract the negative consequences of CF at the behavioral, physiological and neurophysiological levels. CF was triggered by sustained attention demands in the TloadDback, a paradigm that allows inducing comparable levels of CF between individuals, since task demands are adapted to each individual’s cognitive capacity level (Borragán et al., 2017b). Sustained cognitive load-related effects were assessed using subjective fatigue and sleepiness scales, the evolution of accuracy performance within the task, indirect markers of dopaminergic activity (eyeblink and yawning rates), and oxygenation changes (fNIRS) in the cortical mantle during the task and during pre- and post-task resting state periods. Results consistently disclosed CF induction-related effects on all studied parameters. At variance, tDCS had no visible impact on behavioral (performance and subjective scales) and physiological (eyeblink and yawning rates) parameters. These results confirmed by Bayesian evidence (BFs) suggest that the hypothesis that tDCS would exert beneficial effect on behavioral performance in counteracting CF must be rejected, at least within the frame of this experiment. Notwithstanding, the analysis of Cerebral Oxygen Exchange (COE) levels disclosed a tDCS-related inter-hemispheric shift of oxygenation levels during the resting state immediately after the induction of CF, suggesting that tDCS may promote transient compensatory brain activity in response to CF. Furthermore, sleepiness in the tDCS condition increased after a short recovery period (16 min) following the CF-inducing task, suggesting that tDCS stimulation may increase sleepiness at mid-term delay.

### Does tDCS Counteract the Induction of Cognitive Fatigue?

In line with prior findings (Borragán et al., 2017a,b), behavioral results disclosed cognitive load-related increases in CF, paralleled by decreasing performance (accuracy) with time on task. Additionally, triggering CF resulted in decreased arousal as reflected by lower reciprocal reaction times in the PVT, and increased frequency of eye blinks and yawns indirectly reflecting changes in dopamine levels. Furthermore, fNIRS recordings highlighted oxygenation reduction in the dorsal medial prefrontal cortex with decreased performance. These results are in agreement with neuroimaging evidence showing a decrease in neural activity accompanying performance decrease with time on task (Boksem et al., 2005; Lim et al., 2010; Hopstaken et al., 2015). However, our results do not replicate prior studies showing a beneficial effect of tDCS to counter CF and the decline in performance (McIntire et al., 2014; Nelson et al., 2014). Also, tDCS had no visible effect on the evolution of oxygenation levels (COE) during task practice. Several reasons may explain these discrepancies. First, the way we prompted CF was different from prior studies. Indeed, whereas we triggered CF by exposing volunteers to continuous and sustained cognitive demands for a limited period of time, CF was associated with sleep deprivation in the McIntire et al. (2014) study (raising the question of the confounding effect of sleep deprivation-related sleepiness), and Nelson et al. (2014) administered a vigilance task in which the response must be given at spaced intervals in a monotonous setting. It is thus possible that frontal tDCS is less effective to counteract CF-related effects in sustained and high cognitive demands conditions, in which an individual is working at the limits of his cognitive load capacity. Accordingly, it was reported that tDCS on the bilateral posterior parietal cortex decreased accuracy when subjects were performing at high cognitive load (Roe et al., 2015), and that tDCS increased performance only in low performers (Tseng et al., 2012). Second, it must be acknowledged that the reliability of tDCS to induce similar neurophysiological effects between individuals is disputed (Horvath et al., 2015a). A review concluded none or weak evidence for the effects of a single-session tDCS on cognition in healthy participants (Horvath et al., 2015b), and other studies even found negative effects of tDCS on performance (Sandrini et al., 2012). However, this second option is partially contradicted by our finding that tDCS modulates the interhemispheric balance of cerebral oxygen exchange during the resting state following the induction of CF, which is discussed hereafter.

### Does tDCS Modulate Interhemispheric Balance During the Post-CF Resting State?

Oxygen consumption in the left and right hemisphere frontal areas was recorded during three resting state sessions in this experiment, once before and twice after the CF-induction TloadDback task. During pre-task resting state 1 (Rst1), similar levels of oxygenation were observed bilaterally in frontal areas. As mentioned above, cerebral oxygenation decreased with time on task in both hemispheres during practice of the TloadDback task. However, during the second resting state (Rst2) immediately after the TloadDback task, there was a tDCS-related interhemispheric switch in cortical oxygenation. Indeed, higher oxygenation levels were found in the right than in the left hemisphere in the sham tDCS condition (i.e., no actual stimulation). Similarly, tDCS-related changes in interhemispheric balance have been reported in primary motor cortices (Tazoe et al., 2014). To the best of our knowledge, it is the first report of a tDCS-induced inter-hemispheric switch in oxygenation in dorsal prefrontal areas. Noticeably, resting state session Rst2 immediately followed a situation in which brain resources were depleted as the consequence of the continuous demands featured by the TloadDback task. Since no visible inter-hemispheric changes were found at the first resting state Rst1, and tDCS was already applied at that time, it suggests that tDCS effects interacted with the task-related development of CF to impact on subsequent resting state activity. Accordingly, Sun et al. (2014) showed CF-related decreased functional connectivity in the left hemisphere. Altogether, it suggests that the effects of tDCS might be contingent upon the availability of cognitive resources. The functionality of tDCS and CF-related interhemispheric changes in oxygenation immediately after the induction of CF should be investigated in further experiments. Furthermore, at the last resting state (Rst 3) after the recovery period, interhemispheric asymmetry was still present. In addition, correlation analyses suggest that participants who maintained higher levels of activity during this period also experienced higher levels of sleepiness.

### May the Use of tDCS Increase Sleepiness Feelings in the Middle Term?

Even though the application of electrical fluxes to modify brain function dates from already several decades, its extensive use in cognitive science is relatively recent (Nitsche et al., 2008). While there is still an ongoing debate about the real efficacy of tDCS with pro (Reis et al., 2008; Kuo and Nitsche, 2012) and cons (Tremblay et al., 2014; Horvath et al., 2015b) for its effects, evidence is scarce about potential middle- and long-term consequences of its use. In the present work, we investigated middle-term effects of tDCS after a recovery period of 16 min. As shown above, our results disclosed decreased oxygenation levels on the left hemisphere after the recovery period in the Real tDCS condition only. Moreover, participants who kept higher levels of oxygenation during this period reported higher levels of sleepiness at the end. In other words, the presence of higher levels of brain activity during the last resting state (Rst3) session was associated with subsequent increased feeling of sleepiness. In light of these results, we hypothesize that the presence of tDCS might have prevented a suitable recovery during the 16 min break. This observation might be indicating the existence of a trade-off between brain activity and sleepiness, suggesting a negative middle-term effect of tDCS.

While accounting for the distinction between CF and sleepiness is important because both can be considered distinct concepts, this differentiation is often disregarded. The reason is that despite being distinct concepts, fatigue and sleepiness entertain at least partial relationships and are sometimes both present and/or associated in non-restorative sleep conditions (e.g., Ohayon et al., 2005). Therefore, the comparable evolution of CF and sleepiness observed in this experiment is not surprising. Indeed, recent results from our laboratory reveal that discriminating these two states is more likely to take place during the morning hours when homeostatic sleep pressure is minimal (Borragán et al., unpublished).

### Limitations

Transcranial direct current stimulation effects to modulate brain excitability have been quite consistently reported when applied over motor cortices (Nitsche and Paulus, 2000; Kwon et al., 2008; Kirimoto et al., 2011), but results of tDCS over the prefrontal cortex are more unclear (Nikolin et al., 2018). It was proposed that neurophysiological changes should be documented to evidence tDCS effects (Tremblay et al., 2014). In the present experiment, tracking cortical oxygenation changes using NIRS allowed us to quantify the effects of inter-hemispheric changes during the post-task resting state period, which is a positive point. However, our montage centered on prefrontal areas does not allow excluding the contribution of other areas involved in attentional processes such as for instance the parietal cortex. Future studies should thus target more extended networks.

## Conclusion

In this study we showed successful induction of CF after sustained practice on a cognitively demanding task, paralleled with behavioral, physiological and neurophysiological changes. Transcranial DCS (as compared to a sham condition) over the left prefrontal cortex failed to counteract CF-related modifications, possibly because participants were stimulated in a condition in which maximal cognitive resources are recruited to cope with the ongoing task. However, tDCS combined with the induction of CF shifted the interhemispheric oxygenation balance during the post-training resting state. Finally, decreased brain oxygenation after recovery time in the stimulated hemisphere in the tDCS condition was associated with increased self-reported sleepiness. This result suggests that tDCS might actually have been detrimental to recovery from CF during the resting time. Further studies are needed to investigate these issues.

## Author Contributions

GB: data acquisition, analysis, interpretation, drafting, and revising. MG: data acquisition and revising. CG-M: analysis and revising. EDR: data acquisition, analysis, and interpretation. HS: interpretation and revising. PP: analysis, interpretation, drafting, and revising.

## Conflict of Interest Statement

The authors declare that the research was conducted in the absence of any commercial or financial relationships that could be construed as a potential conflict of interest.

## Supplementary Material

FIGURE S1 Indirect physiological indicators of dopamine levels: Eye blinks and Yawning. Total numbers of eye blinks (A) and yawns (B) computed during every resting period of 4 min for each experimental condition. Error bars represents standard errors. Asterisks reflect p-values after Tukey post hoc correction: ^∗^p < 0.05, ^∗∗^p < 0.01, ^∗∗∗^p < 0.001. Only significant differences (p < 0.05) are represented.

TABLE S1 Inclusion criteria. Average, standard deviation, and cut-off established for the inclusion criteria of participants. PSQI, Pittsburgh Sleep Quality Index; HAD, Hospital Anxiety and Depression Scale; ESS, Epworth Sleepiness Scale; FSMC, Fatigue for Motor and Cognitive Functions.
