# Supplementary figures and images for: Transcranial Direct Current Stimulation Does Not Counteract Cognitive Fatigue, but Induces Sleepiness and an Inter-Hemispheric Shift in Brain Oxygenation
Source: Front Psychol. 2018 Nov 30;9:2351. doi: 10.3389/fpsyg.2018.02351 (PMC6284008; doi:10.3389/fpsyg.2018.02351)

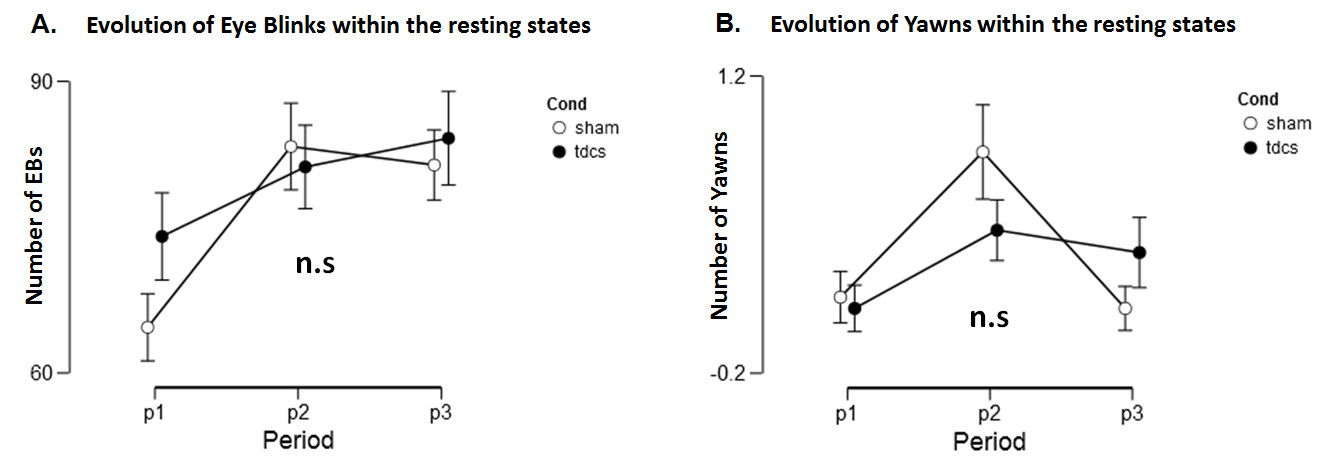

Supplement: FIGURE S1 — Indirect physiological indicators of dopamine levels: Eye blinks and Yawning. Total numbers of eye blinks (A) and yawns (B) computed during every resting period of 4 min for each experimental condition. Error bars represents standard errors. Asterisks reflect p-values after Tukey post hoc correction: ∗p < 0.05, ∗∗p < 0.01, ∗∗∗p < 0.001. Only significant differences (p < 0.05) are represented. [file Image_1.JPEG]

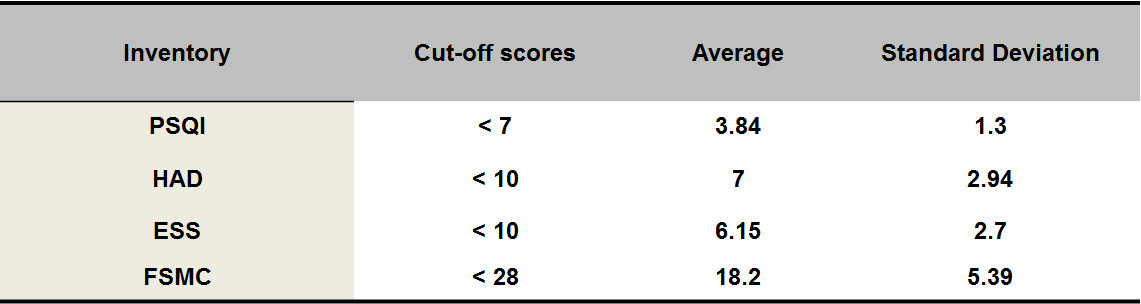

Supplement: TABLE S1 — Inclusion criteria. Average, standard deviation, and cut-off established for the inclusion criteria of participants. PSQI, Pittsburgh Sleep Quality Index; HAD, Hospital Anxiety and Depression Scale; ESS, Epworth Sleepiness Scale; FSMC, Fatigue for Motor and Cognitive Functions. [file Image_2.JPEG]
